# Does Interpersonal Psychotherapy improve clinical care for adolescents with depression attending a rural child and adolescent mental health service? Study protocol for a cluster randomised feasibility trial

**DOI:** 10.1186/1471-244X-7-53

**Published:** 2007-10-04

**Authors:** Cate Bearsley-Smith, Mark Oakley Browne, Ken Sellick, Elmer V Villanueva, Janice Chesters, Karen Francis, Prasuna Reddy

**Affiliations:** 1 School of Psychology, Psychiatry and Psychological Medicine, Monash University, Traralgon, Victoria, Australia; 2 Latrobe Regional Hospital Mental Health Services, Child and Adolescent Program, Traralgon, Victoria, Australia; 3 Department of Rural and Indigenous Health, Monash University, Moe, Victoria, Australia; 4 School of Nursing and Midwifery, Monash University, Churchill, Victoria, Australia; 5 University Department of Rural Health, Flinders and Deakin Universities, Australia

## Abstract

**Background:**

Depression amongst adolescents is a costly societal problem. Little research documents the effectiveness of public mental health services in mapping this problem. Further, it is not clear whether usual care in such services can be improved via clinician training in a relevant evidence based intervention. One such intervention, found to be effective and easily learned amongst novice clinicians, is Interpersonal Psychotherapy (IPT). The study described in the current paper has two main objectives. First, it aims to investigate the impact on clinical care of implementing Interpersonal Psychotherapy for Adolescents for the treatment of adolescent depression within a rural mental health service compared with Treatment as Usual (TAU). The second objective is to record the process and challenges (i.e. feasibility, acceptability, sustainability) associated with implementing and evaluating an evidence-based intervention within a community service. This paper outlines the study rationale and design for this community based research trial.

**Methods/design:**

The study involves a cluster randomisation trial to be conducted within a Child and Adolescent Mental Health Service in rural Australia. All clinicians in the service will be invited to participate. Participating clinicians will be randomised via block design at each of four sites to (a) training and delivery of IPT, or (b) TAU. The primary measure of impact on care will be a clinically significant change in depressive symptomatology, with secondary outcomes involving treatment satisfaction and changes in other symptomatology. Participating adolescents with significant depressive symptomatology, aged 12 to 18 years, will complete assessment measures at Weeks 0, 12 and 24 of treatment. They will also complete a depression inventory once a month during that period. This study aims to recruit 60 adolescent participants and their parent/guardian/s. A power analysis is not indicated as an intra-class correlation coefficient will be calculated and used to inform sample size calculations for subsequent large-scale trials. Qualitative data regarding process implementation will be collected quarterly from focus groups with participating clinicians over 18 months, plus phone interviews with participating adolescents and parent/guardians at 12 weeks and 24 weeks of treatment. The focus group qualitative data will be analysed using a Fourth Generation Evaluation methodology that includes a constant comparative cyclic analysis method.

**Discussion:**

This study protocol will be informative for researchers and clinicians interested in considering, designing and/or conducting cluster randomised trials within community practice such as mental health services.

**Trial Registration:**

Australian Clinical Trials Registry ACTRNO12607000324415

## Background

The immediate and long-term individual, interpersonal and societal costs of depression are substantial [1]. Up to one in five people will have suffered the effects of major depression before the age of 18 years [2]. Despite limited research regarding depression amongst rural adolescents, we know that rural young men are at particularly high risk of suicide compared to their metropolitan peers [3]. Depression during adolescence typically complicates progression through the essential developmental tasks of that time leading to secondary difficulties [4,5]. To address such problems, effective and accessible treatment is needed. When detected, however, treatment provided for depression is often inconsistent with evidence-based treatment guidelines [6]. Whether or not such treatment discrepancy is problematic for adolescent outcomes remains unclear.

Questions remain regarding the most effective community treatment for adolescents with depression. Antidepressant medication continues to be associated with controversy regarding its efficacy and safety in use with adolescents [7]. Structured psychological interventions, particularly Cognitive Behaviour Therapy (CBT) and Interpersonal Psychotherapy (IPT), have demonstrated efficacy for the treatment of adolescent depression [8,9]. These psychotherapies are also used as treatments alongside medication, or in the maintenance of post depressive episodes.

Efficacy studies, requiring strict treatment and control conditions, typically exclude clients with complex clinical presentations. In reality, adolescents with depression frequently present with comorbid mental or substance-use disorders [10]. Effectiveness trials are needed to investigate the feasibility and value of implementing structured psychological interventions in community practice.

The available evidence suggests IPT is an efficacious treatment for depression [11] which can be easily learned by Australian mental health professionals [12] and other novice therapists [13,14]. IPT is based on the formulation that relationships are inherently inter-related with emotional wellbeing. The therapy aims to decrease depressive symptomatology and improve the relational functioning of clients. The treatment focuses on a key interpersonal problem area identified for the individual (e.g. grief, interpersonal role disputes, interpersonal role transitions). IPT is manual-based and time-limited and can be administered effectively with or without medication [15].

From a systematic review of IPT efficacy in the treatment of depressive spectrum disorders between 1974 and 2002, a meta-analysis of 13 studies found IPT superior in efficacy to placebo for mean improvement in depressive symptoms (weighted mean difference [WMD] = -3.57; 95% confidence interval [CI] -5.98, -1.16) and lower short-term drop out rate (relative risk [RR] = 0.59; 95% CI 0.36, 0.99). IPT was similar in efficacy to medication and did not increase when combined with medication. IPT performed significantly better than CBT for mean difference improvement (WMD = -2.16; 95% CI -4.16, -0.15) though not for total remission [16].

Interpersonal Psychotherapy for Adolescents (IPT-A) has evolved from the adult version (IPT), making adaptations for the developmental issues of adolescents. Amongst adolescents with depression, IPT-A has been demonstrated to be superior to Treatment as Usual (TAU) in an effectiveness study of five school-based mental health clinics in New York [17]. In a trial with Puerto Rican adolescents, remission of depressive symptoms was achieved by 82% of adolescents receiving IPT-A and 59% receiving CBT. Compared to the waiting list control, those in an IPT-A group showed increased self-esteem and social adaptation [18]. IPT-A has also demonstrated effectiveness in the treatment of moderate to severe depression in adolescents by well-supervised novice IPT-A therapists [13].

IPT appears a promising intervention to trial for the treatment of adolescents with depression attending a rural Child and Adolescent Mental Health Services (CAMHS). CAMHS aims to treat adolescents presenting with severe, usually comorbid, mental health difficulties. In rural areas this is complicated by limited availability of mental health specialists. IPT appears particularly valuable in this context given its appeal to clinicians and easy delivery by a broad range of mental health professionals. The TAU of adolescent depression in CAMHS is, on contrast, poorly defined and of unknown quality or effectiveness.

The current project explores the impact of implementing IPT in a rural CAMHS and the feasibility of comparing IPT and TAU in practice. Given that implementation and evaluation of interventions in health services is typically complex, a qualitative process evaluation will be incorporated to explore the contextual factors associated with the implementation [19].

The objectives of the clinical research are:

1. to investigate the impact on clinical care of implementing IPT for the treatment of adolescent depression within a rural CAMHS compared with TAU.

2. to evaluate the issues associated with implementing a cluster randomised clinical trial in CAMHS (i.e. feasibility, acceptability, sustainability).

## Methods/Design

### Sample

The research is being conducted within the LRH CAMHS in rural Victoria, Australia. The service provides care for children and adolescents across 40,000 square kilometres with a population of 236,000. All clinicians, employed full- or part-time within the LRH CAMHS, other than the principle investigator, are invited to participate. At the time of writing this includes 18 clinicians based at four sites. The clinicians comprise psychologists, social workers, occupational therapists, mental health nurses and a psychiatry registrar. The trial aims to include a minimum of 60 adolescent clients of LRH CAMHS, aged 12 to 18 years inclusive, to be recruited over 12 months. The sample size was determined on the basis of logistic feasibility and resource constraints. In addition, given the lack of information about the likely size of the intra-class correlation coefficient (ICC), one intention of this study is to estimate this statistic to assist in sample size calculation for a larger subsequent effectiveness trial [20].

### Inclusion/Exclusion criteria

Adolescents are eligible to participate if (a) they meet the clinical cut-off for significant depressive symptomatology in the Reynold's Adolescent Depression Scale – 2^nd ^Edition (RADS-2) (T ≥ 61) [21], or (b) receive a DSM-IV-TR [22] or ICD-10 [23] diagnosis of a depressive disorder (i.e. Major Depressive Disorder (MDD), Dysthymic Disorder, Depressive Disorder NOS, or Adjustment Disorder with Depressed Mood) as affirmed by the team psychiatrist, and (c) have a Children's Global Assessment Scale (CGAS) [22] of 65 or lower. Clients will need parental/guardian consent to participate and the agreement of the CAMHS consultant psychiatrist. Adolescents will not be included if they are actively suicidal, have a life-threatening condition, psychosis or schizophrenia. Parents/guardians are eligible to participate if their adolescent child is participating and they have no exclusion criteria.

### Design

Approval for the research has been given by the Latrobe Regional Hospital (LRH) Human Research Ethics Committee (HREC) and the Monash University Standing Committee on Ethics in Research Involving Humans (SCERH).

#### Randomisation

A cluster randomisation design was chosen over simple client randomisation to overcome the obstacles of randomizing adolescents to treatment streams (i.e. to specific clinicians). Randomisation of adolescents could not be assured given limitations on the availability of specific clinicians at any given time and site.

The clinical assignment allocation process involves block randomisation of participating clinicians within each of the four sites to either IPT training or TAU. Allocation of clients to clinicians continues as per usual practice of availability. Randomisation was conducted centrally.

#### Treatments

The IPT stream comprises 12 weekly manual-based sessions with the adolescent. The TAU stream comprises usual care for the client. This may involve, for example, individual counseling, family work, liaison with schools and other professions, psychopharmacology, etc. In both streams the clinician will record monthly the treatment strategies used, the frequency of sessions, any additions or changes in medication, and any critical incidents which have occurred.

#### Concurrent process evaluation

Implementation issues will be investigated qualitatively using quarterly focus groups with clinicians and through individual telephone interviews with adolescent participants and their parent/guardians after receiving 12 weeks and 24 weeks of treatment. The telephone interviews with participating adolescents and parent/guardians will follow a semi-structured schedule to guide the interview. The questions will explore the participant's views on the treatment provided. Example questions include, 'How effective do you feel the treatment has been that you have received at CAMHS?', 'What have been the most helpful components of the treatment?' and 'What changes would you recommend in the treatment you have received at CAMHS?' The interview will take approximately 20 minutes.

A preliminary action research project was conducted with the LRH CAMHS clinicians to encourage clinician input and engagement in the design of the project. This comprised focus groups conducted with clinicians between three and five occasions during 2006. This process helped to shape the research to the clinical environment, and the clinical environment to research involvement.

### Main outcome measures

Monitoring and outcome assessment will use reliable and valid measures applied in similar research. These include adolescent self-report, parent-report and clinician ratings:

#### 1. Clinician-rated diagnostic status and clinical severity

a. Health of the National Outcome Scales (HoNOSCA) [24]. This is a global measure of child and adolescent mental health status. It is a standard CAMHS outcome measure used to rate changes in CAMHS client behaviour, impairment, symptoms, and social functioning.

b. CGAS [22]. The CGAS, presented in the DSM-IV-TR, is a clinician-rating scale (1 through 100) used rate the general functioning of children and adolescents.

c. Treatment Recording Sheet (TRS). This clinician-report record was adapted with CAMHS clinician input from the Hawaii Child and Adolescent Mental Health Division (CAMHD) Intervention Strategies Codesheet and Codebook [25]. It is a monthly record of specific intervention strategies used with an adolescent. It also records any critical incidents or medication changes occurring for the adolescent during that month. Its development is discussed elsewhere.

#### 2. Self-report

a. RADS-2 [21]. This adolescent-report measure takes 5 to 10 minutes for an adolescent to complete and has a Grade 3 reading level. It is a screening measure for adolescent depression, providing a clinical cut-off for ratings of mild, moderate and severe depressive symptomatology as well as six critical items for clinical consideration. It shows good reliability and validity.

b. Strengths and Difficulties Questionnaire (SDQ-S) [26]. The SDQ-S is a standard outcome measure administered in CAMHS. It is a brief behavioural screening questionnaire for children and adolescents. It includes items related to emotional, conduct, and hyperactivity/inattention symptoms, peer problems and prosocial behaviour.

c. Multidimensional Adolescent Satisfaction Scale (MASS) [27]. The MASS is a 21-item scale measuring adolescents satisfaction with various dimensions of mental health service treatment. It has good reliability and demonstrated convergent, discriminate, and predictive validity. Its subscales include counselor qualities (perceived competence of the clinician and quality of the relationship), meeting needs (perceived adequacy of quantity and type of service received), effectiveness (perceived benefit of service), and counselor conflict (experience of negative interactions with counselor).

d. Client Satisfaction Questionnaire (CSQ-8) [28]. The 8-item CSQ-8 consists assesses overall level of satisfaction with the treatment received.

#### 3. Parent-report

a. Strengths and Difficulties Questionnaire (SDQ-P) [26]. The SDQ-P is the parent-version of the SDQ, as described above

b. Short Mood and Feelings Questionnaire – Parent Version (SMFQ-P) [29]. The SMFQ-P is a 5 minute 13-item uni-dimensional parent-report measure of their child/adolescent's depressive symptomatology. It shows good internal reliability and discriminated between psychiatrically referred and non-referred children.

c. Client Satisfaction Questionnaire (CSQ-8) [28]. This measure is described above.

#### 4. Other

Demographic details of clients. Age, gender, previous mental health treatment, medication, parental occupation, family members in home, and ethnicity were recorded.

#### 5. Process evaluation

Evaluation questionnaires will be administered to all clinicians regarding use of the TRS at onset of research involvement and after 18 months. Evaluation of the IPT training will be conducted with IPT-trained clinicians immediately pre- and post-training and at 18 months follow-up. Focus groups conducted quarterly with clinicians will explore (a) contextual information regarding the research implementation (to assist interpretation of the quantitative trial results [19]), and (b) the feasibility, acceptability and sustainability of conducting the clinical research in CAMHS. Individual telephone interviews with adolescent participants and their parent/guardians will explore their satisfaction with treatment. Responses will be audio-tape recorded and transcribed.

## Analysis of results

### Quantitative analysis

Analysis will adopt an intention-to-treat design. The primary quantitative outcome measure will be change in depressive symptomatology. This will be evaluated as (a) the proportion of clients per treatment group with remission of depressive symptoms, defined as RADS-2 score below the clinical cut-off; (b) the proportion of clients per treatment group with clinical improvement of depressive symptoms, defined as having a 50% or higher reduction of depressive symptoms; (c) the difference in mean depressive symptoms post-treatment. The secondary quantitative outcome measure will be group differences in adolescent and parent/guardian satisfaction with treatment at 12 weeks and 24 weeks after start of treatment. Treatment acceptability will also be measured by proportion of clients per treatment group who do not complete treatment [16].

The quantitative analyses will compare group differences (i.e. remission, clinical improvement, mean difference, and non-completion) between the IPT and TAU groups. These analyses will allow estimation of the effect size of IPT on these outcomes. Comparison will be made between baseline and outcomes at 12 weeks and 24 weeks from start of treatment. Our main aim is one of estimation rather than hypothesis testing. Thus, effect sizes with 95% confidence intervals will be calculated in favour of significance testing in order to provide information for subsequent trials.

### Qualitative analysis

The qualitative data will be analysed using a Fourth Generation Evaluation methodology [30] that includes a constant comparative cyclic analysis method. This involves initial scanning of the data sets, development of themes from the data sets, comparison of analysed data across the data sets and formulation of final recommendations for the improvements in similar CAMHS research trials.

## Discussion

This protocol is the result of collaboration between a rural CAMHS and Monash University. It developed from an interest within CAMHS of implementing and evaluating a focused evidence-based practice clinic. The key interests of CAMHS and academic collaborators are on the evaluation of practice and recording the process issues associated with implementation of a clinical trial in CAMHS. The study aims to inform development of a larger scale definitive clinical trial.

The challenges of linking research and practice in health services are well documented. With the increased pressure on services to deliver and document evidence-based practice, the issues concerning integration of evidence and practice are increasingly important. This study seeks to look at these process issues arising in a rural CAMHS embarking on a clinical trial.

A key aim of the project trial is to assess the feasibility of conducting effectiveness trials of short-term psychological interventions in a rural CAMHS. To assess issues of feasibility and to aid interpretation of trial results, a combination of qualitative and quantitative methodologies will be applied. The facilitated discussion groups with clinicians, and interviews with adolescent and parent/guardian participants, plus a research diary, aim to capture the process issues as they arise and any related complexities and solutions uncovered during the trial. These findings are considered important to assist further mental health services researchers in the design of similar future ventures. They are also important in a political environment of increasing evidence-based practice pressure. Documentation of the implementation obstacles and facilitators can practically inform discussions, expectations and decisions in this area.

To fit in with the CAMHS realities, a cluster randomisation design was chosen over client randomisation. The consideration of client randomisation raised concerns for both clinicians and researchers. It was not feasible to do client randomisation given the scarcity of clinicians based at each of the four rural sites. At any given time a clinician from the allocated treatment stream may not be available at that site to work with the adolescent. For example, one site may have only one participating clinician and others only one for each treatment stream. Cluster randomisation provided the solution to this dilemma. It has the advantage of allowing usual-care allocation of clients and would provide an estimate of the ICC which would assist sample size planning in a larger-scale subsequent trial, if judged to be feasible and valuable.

Given the uncertainties existing regarding the safety and efficacy of psychopharmacological treatment of adolescents [7], it is important that effective psychological interventions are readily available for the treatment of adolescent depression. IPT appears a useful intervention for this purpose, given that it is described as easily learned and applied by a range of mental health professionals [13,14].

The study of TAU, however, is also an important component of the study. First, there has been little study of what TAU specifically entails in a multidisciplinary rural mental health service. Clarification of the content of this is important if we are to understand or evaluate it or compare it to alternative forms of treatment. Second, the effectiveness of TAU is unknown. If TAU proves as effective in practice as identified evidence-based practices then questions about the value of implementation efforts regarding the latter may be raised. Definitive answers about effectiveness of TAU compared to IPT in practice will, however, require large sample sizes and replication of results with different studies, a challenging endeavor within community practice.

The real-world context of this study means that it faces important limitations. First, key variables cannot be controlled in this environment. The methodology is impacted by such factors as clinician turn-over, mixed attitudes to involvement in research, questions of treatment fidelity, challenges of data collection, amongst others. The decision was made, therefore, to describe the variables rather than attempt to control.

A cluster randomisation design was utilized to avoid the inevitable implementation failures that would occur randomising individual participants to particular treatment streams. Randomisation of individuals would not be possible when, for example, the relevant clinician at that site was unavailable to take another case. Instead it was decided that cases would be allocated by the team. This lack of allocation blinding and randomisation of participants is likely to introduce bias to the allocation of cases. Nevertheless, the cluster randomisation design is likely to detect and account for the variability between groups. The limitations found during this research will have important findings. They will illustrate sources of complexity in seeking to marry evidence and practice in child and mental health services.

The transferability of efficacious treatments into practice is an important issue for clinical researchers and clinicians. It is a complex area of implementation challenges. This article describes the methodological responses to challenges foreseen in conducting a clinical feasibility trial concurrently being conducted within a rural CAMHS. This study protocol illustrates a collaborative clinical research design of potential value to researchers interested in innovative and realistic treatment trials in this area.

## Competing interests

The author(s) declare that they have no competing interests.

## Authors' contributions

CBS oversaw development and implementation of the research design and drafted the current manuscript. All authors contributed to the research design, and read and approved the final manuscript.

## Pre-publication history

The pre-publication history for this paper can be accessed here:



## References

[B1] Greenberg PE, Stiglin LE, Finkelstein SN, Berbdt ER (1993). The economic burden of depression in 1990. Journal of Clinical Psychiatry.

[B2] Hankin BL (2006). Adolescent depression: description, causes, and interventions. Epilepsy Behav.

[B3] Caldwell TM, Jorm AF, Dear KBG (2004). Suicide and mental health in rural, remote and metropolitan areas in Australia. MJA.

[B4] Lewinsohn PM, Rohde P, Klein DN, Seely JR (1999). Natural course of adolescent major depressive disorder: I.  continuity into young adulthood. Journal of American Academy of Child and Adolescent Psychiatry.

[B5] Weissman MM, Wolk S, Goldstein RB, Moreau D, Adams P, Greenwald S, Klier CM, Ryan ND, Dahl RE, Wickramaratne P (1999). Depressed adolescents grown up. Journal of American Medical Association.

[B6] Wang PS, Berglund P, Kessler RC (2000). Recent care of common mental disorders in the United States:  prevalence and conformance with evidence-based recommendations. Journal of General Internal Medicine.

[B7] Cipriani A, Barbui C, Geddes JR (2005). Suicide, depression, and antidepressants. BMJ.

[B8] Beutler LE, Machado PPP, Neufeldt SA, Bergin AE, Garfield SL (1994). Therapist variables. Handbook of Psychotherapy and Behavior Change.

[B9] Roth A, Fonagy P (1996). What Works for Whom?  A Critical Review of Psychotherapy Research.

[B10] Tonge B (1998). Depression in Young People. Australian Prescriber.

[B11] Kotova E (2005). A meta-analysis of Interpersonal Psychotherapy. Dissertation Abstracts International: Section B: The Sciences and Engineering.

[B12] Reay R, Stuart S, Owen C (2003). Implementation and effectiveness of interpersonal psychotherapy in a community mental health service. Australasian Psychiatry.

[B13] Santor DA, Kusumakar V (2001). Open trial of interpersonal therapy in adolescents with moderate to severe major depression:  Effectiveness of novice IPT therapists. J Am Acad Child Adolesc Psychiatry.

[B14] Rounsaville BJ, Chevron ES, Weissman MM, Prusoff BA, Frank E (1986). Training therapists to perform Interpersonal Psychotherapy in clinical trials. Comprehensive Psychiatry.

[B15] Weissman MM, Markowitz JC, Klerman GL (2000). Comprehensive Guide to Interpersonal Psychotherapy.

[B16] de Mello MF, de Jesus Mari J, Bacaltchuk J, Verdeli H, Neugebauer R (2005). A systematic review of research findings on the efficacy of interpersonal therapy for depressive disorders. European Archives of Psychiatry and Clinical Neuroscience.

[B17] Mufson L, Dorta KP, Wickramaratne P, Nomura Y, Olfson M, Weissman MM (2004). A randomized effectiveness trial of interpersonal psychotherapy for depressed adolescents. Arch Gen Psychiatry.

[B18] Rossello J, Bernal G (1999). The efficacy of cognitive-behavioral and interpersonal treatments for depression in Puerto Rican adolescents. J Consult Clin Psychol.

[B19] Oakley A, Strange V, Bonell C, Allen E, Stephenson J (2006). Process evaluation in randomised controlled trials of complex interactions. British Medical Journal.

[B20] Lobban F, Gamble C, Kinderman P, Taylor L, Chandler C, Tyler E, Peters S, Pontin E, Sellwood W, Morriss RK (2007). Enhanced relapse prevention for bipolar disorder - ERP trial.  A cluster randomised controlled trial to assess the feasibility of training care coordinators to offer enhanced relapse prevention for bipolar disorder. BioMed Central Psychiatry.

[B21] Reynolds WM (2002). Reynold's Adolescent Depression Scale - 2nd Edition.

[B22] First MB, APA (2000). Diagnostic and Statistical Manual of Mental Disorders - Fourth Edition - Text Revision (DSM-IV-TR).

[B23] Janca A, Ustun TB, Drimmelen J, Dittmann V, Isaac M (1994). ICD-10 Symptom Checklist for Mental Disorders, Version 1.1.

[B24] Gowers SG, Harrington RC, Whitton A, Lelliott P, Wing J, Beevor A, Jezzard R (1999). A brief scale for measuring the outcomes of emotional and behavioural disorders in children:  HoNOSCA. Br J Psychiatry.

[B25] Daleiden EL, Lee J, Tolman R, Division. HDHCAMH Annual Evaluation Report Fiscal Year 2004.

[B26] Goodman R (2001). Psychometric properties of the Strengths and Difficulties Questionnaire (SDQ). J Am Acad Child Adolesc Psychiatry.

[B27] Garland AF, Saltzman M, Aarons G (2000). Adolescent satisfaction with mental health services: development of a multi-dimensional scale. Eval Program Plann.

[B28] Attkisson CC, Zwich R (1982). The client satisfaction questionnaire.  Psychometric properties and correlations with service utilization and psychotherapy outcome. Eval Program Plann.

[B29] Angold A, Costello EJ, Messer EC, Pickles A (1995). Development of a short questionnaire for use in epidemiological studies of depression in children and adolescents. Int J Meth Psychiatric Res.

[B30] Guba E, Lincoln Y (1989). Fourth Generation Evaluation.

